# Bcl-XL but Not Bcl-2 Is a Potential Target in Medulloblastoma Therapy

**DOI:** 10.3390/ph15010091

**Published:** 2022-01-14

**Authors:** Mike-Andrew Westhoff, Marie Schuler-Ortoli, Daniela Zerrinius, Amina Hadzalic, Andrea Schuster, Hannah Strobel, Angelika Scheuerle, Tiana Wong, Christian Rainer Wirtz, Klaus-Michael Debatin, Aurelia Peraud

**Affiliations:** 1 Department of Pediatrics and Adolescent Medicine, Ulm University Hospital, 89075 Ulm, Germany; andrea.schuster@uniklinik-ulm.de (A.S.); hannah.strobel@uniklinik-ulm.de (H.S.); bianca.welz@uniklinik-ulm.de (T.W.); klaus-michael.debatin@uniklinik-ulm.de (K.-M.D.); 2 Section Pediatric Neurosurgery, Department of Neurosurgery, Ulm University Hospital, 89081 Ulm, Germany; marie.schuler-ortoli@uniklinik-ulm.de (M.S.-O.); daniela.zerrinius@uniklinik-ulm.de (D.Z.); amina.hadzalic@uniklinik-ulm.de (A.H.); 3 Department of Neuropathology, Ulm University, 89312 Guenzburg, Germany; angelika.scheuerle@uniklinik-ulm.de; 4 Department of Neurosurgery, Ulm University Hospital, 89081 Ulm, Germany; verena.mack@uniklinik-ulm.de

**Keywords:** medulloblastoma, apoptosis, Bcl-2, Bcl-XL, ABT-199 (Venetoclax), ABT-263 (Navitoclax), chemotherapy

## Abstract

Medulloblastoma (MB) is the most common solid tumour in children and, despite current treatment with a rather aggressive combination therapy, accounts for 10% of all deaths associated with paediatric cancer. Breaking the tumour cells’ intrinsic resistance to therapy-induced cell death should lead to less aggressive and more effective treatment options. In other tumour entities, this has been achieved by modulating the balance between the various pro- and anti-apoptotic members of the Bcl-2 family with small molecule inhibitors. To evaluate the therapeutic benefits of ABT-199 (Venetoclax), a Bcl-2 inhibitor, and ABT-263 (Navitoclax), a dual Bcl-XL/Bcl-2 inhibitor, increasingly more relevant model systems were investigated. Starting from established MB cell lines, progressing to primary patient-derived material and finally an experimental tumour system imbedded in an organic environment were chosen. Assessment of the metabolic activity (a surrogate readout for population viability), the induction of DNA fragmentation (apoptosis) and changes in cell number (the combined effect of alterations in proliferation and cell death induction) revealed that ABT-263, but not ABT-199, is a promising candidate for combination therapy, synergizing with cell death-inducing stimuli. Interestingly, in the experimental tumour setting, the sensitizing effect of ABT-263 seems to be predominantly mediated via an anti-proliferative and not a pro-apoptotic effect, opening a future line of investigation. Our data show that modulation of specific members of the Bcl-2 family might be a promising therapeutic addition for the treatment of MB.

## 1. Introduction

Medulloblastoma (MB) is the most common solid tumour in children and accounts for approximately 10% of all childhood cancer deaths [1]. The current standard of care is a rather aggressive multimodal therapeutic regimen, including microsurgical resection, chemotherapy and radiotherapy; however, overall patient survival is still greatly impeded, with some ethnical groups faring significantly worse than others [2]. Importantly, MB must be considered an umbrella term for a collection of rather diverse subtypes of tumours, with varying degrees of therapeutic responses. The current consensus given in the last WHO classification of central nervous system (CNS) tumours from 2016 has addressed four MB molecular subgroups, termed wingless (WNT), sonic hedgehog (SHH) with or without TP53 activation, Group 3 and Group 4, which are non-WNT/non-SHH activated [3]. WNT-activated, SHH-activated and the non-WNT/non-SHH MBs can be differentiated by several immunohistochemical markers, namely β-Catenin, Yap1, p75NGFR, Otx2 and p53 [4,5]. The most favourable prognosis is associated with the activation of the WNT signalling pathway, which is unfortunately also the rarest. Group 3, in contrast, has the worst prognosis and accounts for 25% of all MBs. The SHH and Group 4 tumours generally exhibit an intermediate prognosis, while the presence of mutated TP53 is a prognostic marker for poor survival in all subgroups [6].

There are two major obstacles to the implementation of successful new treatment strategies of MB. One, treatment, as with many paediatric tumours, is often associated with long-term sequelae, such as hormonal derangements, growth retardation, neurocognitive decline and secondary malignancies, as well as psychological problems in patients and families [7,8]. Two, the clinical evaluation of novel therapies is often hindered by the relative low numbers of cases that can be recruited. While new trial designs have, to a certain extent, alleviated this problem for some paediatric cancers, the distinct genetic and epidemiological differences in the subgroups of MB severely limit the options with this particular set of malignancies [7,8]. We, therefore, wanted to find a treatment strategy that addresses these two points, reducing the need for high concentrations of radio- and chemotherapy, thus lowering the risk of long-term side effects and having a relatively broad applicability independent of subtypes. In their landmark publications, Hanahan and Weinberg identified resistance to cell death as a defining feature, a so-called hallmark, of cancer and suggest the use of BH3 mimetics which target members of the Bcl-2 family to overcome this resistance [9,10].

The members of the Bcl-2 family are defined as containing at least one Bcl-2 homology (BH) domain and are classified as either pro- or anti-apoptotic. Their balance is decisive of cell survival or death [11], i.e., they are gatekeepers of the integrity of the mitochondrial outer membrane and thereby apoptosis induction. The BH3-only Bcl-2 family members are a large group of pro-apoptotic proteins that bind and, thus, inhibit anti-apoptotic Bcl-2 family members [12]. There are several reasons why the Bcl-2 family is an attractive target for tumour therapy [10]. The apoptosis pathway is a natural barrier to cancer development, which in turn means that cancer cells will almost invariably display some sort of dysregulation in this signalling network. Bcl-2 proteins are mediators of the so-called ‘‘apoptotic trigger’’ and as such act as a central hub that integrates many diverse death signals. As such, they are, for example, downstream of the most commonly inactivated protein in cancer, p53, so targeting the Bcl-2 family bypasses this block in signalling. In addition, the Bcl-2 family also serves as a molecular link between the (inactivated) apoptosis pathway and a second survival strategy exhibited by many tumours, the autophagy network. Finally, therapeutic targeting of Bcl-2 proteins with BH3 mimetics, while not without side effects, has displayed a rather favourable safety profile [13,14].

BH3 mimetics are small peptides that mimic the BH3 domain of these proteins and thus can facilitate cell death. ABT-737 (Obatoclax) and ABT-263 (Navitoclax), two exemplary BH3 mimetics, efficiently induce apoptosis as single agents in leukaemia and lung cancer [13,14]. Furthermore, preclinical studies of combination therapies with classical cytotoxic agents have shown promising results, even in cancers less sensitive to single agent treatment, e.g., B-cell lymphoma [14,15]. Our own work also suggest that these peptides could be efficiently used in glioblastoma therapy [16,17,18], but little work has so far been done in MB.

## 2. Materials and Methods

### 2.1. Cells and Growth Conditions

HD-MB 03 cells were purchased from DSMZ (Heidelberg, Germany), while D425, D458 and Daoy cell lines were a kind gift from Simone Fulda (Goethe University, Frankfurt, Germany) and RS4;11 cells were a kind gift from Lüder Hinrich Meyer (University Hospital Ulm, Ulm, Germany). The D425 and D458 cells were cultured in Improved Minimum Essential Medium (MEM) Zinc Option supplemented with 20% foetal calf serum (FCS), 1% Pen/Strep, 1% MEM Non-Essential Amino Acids Solution (100×) (NEAA) (all from Gibco, Life Technologies, Grand Island, NY, USA) and 2.5% HEPES-Buffer (Biochrom AG, Berlin, Germany). Daoy cells were cultured in Minimum Essential Medium (MEM) supplemented with 20% FCS, 1% Pen/Strep, 1% NEAA (100×), 1% L-glutamine, 1% Sodium Pyruvate (100 mM) (all from GibcoLife Technologies) and 2.5% HEPES-Buffer (Biochrom AG, Berlin, Germany). HD-MB 03 cells were cultured in RPMI Medium 1640 supplemented with 10% FCS, 1% Pen/Strep (all from GibcoLife Technologies, Grand Island, NY, USA) and 2.5% HEPES-Buffer (Biochrom AG, Berlin, Germany). RS4; 11 cells were cultured in RPMI Medium 1640 supplemented with 20% FCS, 1% Pen/Strep and 1% L-glutamine (all from from GibcoLife Technologies, Grand Island, NY, USA).

### 2.2. Primary Patient Material

Patient-derived MB spheres were grown from surgical specimens, after patients’ or guardians’ consent was obtained. The previously published protocol for glioblastoma patient material [12,19] was adapted for this purpose. Specimen #322 was minced and taken up in ice-cold PBS, followed by 5 min centrifugation at 12,000 rpm at room temperature. After discharging the liquid, the tumour pellet was taken up in 5 mL TrypLE Express (Gibco, Life Technologies, Grand Island, NY, USA) for 5 min, followed by filtration through a sieve (pore size, 70 µm) and taken up in DMEM/F-12 (HAM) medium (Gibco, Life Technologies, Grand Island, NY, USA), supplemented with L-glutamine, 20 µg/mL epidermal growth factor (EGF; Biomol GmbH, Hamburg, Germany), 10 µg/mL basic fibroblast growth factor (bFGF; Miltenyi Biotec GmbH, Bergisch Gladbach, Germany) and 2% B27 supplement (Gibco, Life Technologies, Grand Island, NY, USA). Cells were cultured as free-floating spheres in non-adhesive plastic flasks. For culturing the primary differentiated cells, different types of media and supplements were tested. Cells grew best in the formulation also used for Daoy cells. Cells were differentiated by seeding on tissue culture-treated adhesive plastic in the presence of FCS and passaged a maximum of ten times. The study was approved by the Ethics Committee, Medical Faculty, Ulm University.

### 2.3. Reagents

ABT-199 and ABT-263 were purchased from Selleckchem (Houston, TX, USA) and dissolved in dimethyl sulfoxide (DMSO). Doxorubicin and vincristine were obtained from the central pharmacy from Ulm University Medical Center (Ulm, Germany). Both chemotherapeutic agents were stored at 4°C for a maximal duration of six weeks.

- Concentrations used for established cell lines:
  ABT-199 10 nM
  ABT-263 1 µM
  Doxorubicin 5 nM (Daoy) or 10 nM (all others)
  Vincristine 1 nM (Daoy) or 0.5 nM (all others)
  Growth factor reduction: 1.5% FCS (Daoy) or 5% FCS (the others)
- Concentrations used for primary patient-derived cells:
  ABT-199 10 nM
  ABT-263 1 µM
  Doxorubicin 5 nM and 10 nM (lower concentration for subsequent experiments)
  Vincristine 0.5 nM and 1 nM (lower concentration for subsequent experiments)
  Growth factor reduction: 50% of original concentration for primary cells,
  25% of original concentration for their differentiated progeny.

### 2.4. Cell Viability Assay

In order to examine cellular viabilities, 3-[4,5-Dimethythiazol-2-yl]-2,5-diphenyl tetrazolium bromide (MTT)-based assays were performed, as previously described [19]. Here, the viability (metabolic activity) of a cell population is determined via a colorimetric assay for assessing cell metabolic activity, based on the relative activity of NAD(P)H-dependent cellular oxidoreductase enzymes. For this assay, cells were seeded in a 96-well flat-bottomed tissue culture plate and allowed to settle overnight. The solvent DMSO corresponding with the highest inhibitor concentration was defined as control. To assess the metabolic activity, the medium of each well was aspirated, and 100 µL of 1× MTT solution (Sigma-Aldrich, Hamburg, Germany) were added to each well and incubated for up to 3 h at 37 °C. For the experiments with the non-adherent stem-like cells, 20 µL of 5× MTT stock solution were directly added to each well and incubated for up to 3 h at 37 °C. The reaction was stopped by adding 100 µL 100% isopropanol alcohol in each well. The optical density (OD) of the resulting solution was determined with an automated microplate reader (ELx800; BioTek and Tecan infinite pro) at a wavelength of 550 nm. Relative viability was expressed as (ODstimulation − ODblank)/(ODcontrol − ODblank).

### 2.5. Western Blot Analysis

Protein expression was determined by Western blot analysis, as previously described [12]. The following antibodies were used: anti-Mcl-1 (1:1000; #559027, BD Pharmingen, Franklin Lakes, NJ, USA), anti-Bcl-2 (1:1000; #551098, BD Pharmingen, Franklin Lakes, NJ, USA), anti-Bad (1:1000; #9292, Cell Signalling, Frankfurt, Germany), anti-Bax (1:1000; #2772, Cell Signalling, Frankfurt, Germany), anti-Bcl-xL (1:1000; #610211, BD Pharmingen, Franklin Lakes, NJ, USA), anti-Bim (1:1000, #2819, Cell Signalling, Frankfurt, Germany), anti-β-actin (1:10000; #A5441, Sigma-Aldrich, St. Louis, MO, USA). Secondary HRP-linked antibodies were purchased from Jackson ImmunoResarch (#115-035-006, #111-035-006, West Grove, PA, USA).

### 2.6. Changes in Cell Number (‘CASY’)

The cells were counted using CASY1 DT (Roche Diagnostics, Indianapolis, IN, USA), as previously described [20]. Cells were seeded in 24-well plates and allowed to settle overnight at 37 °C. This was followed by stimulation with the indicated substances and combinations thereof. At the appointed time, the supernatant was aspirated, and cells were detached with 300 µL of Trypsin/EDTA. The resuspended cells were then diluted in a ratio of 1:100 in CASYton solution, and the cell number was measured using CASY1 DT, which uses the current exclusion method based on the conductivity and size of cells varying between living and dead cells. An exception was made for primary patient-derived spheres, as growth was non-adherent. Here, spheres were dispersed through vigorous pipetting and diluted directly from medium into CASYton solution.

### 2.7. Determination of Apoptosis

As a surrogate readout for apoptotic cell death, DNA fragmentation in propidium iodide (PI)-stained nuclei was cytometrically evaluated, as previously described [20]. Cells were seeded in 24-well plates and allowed to settle overnight at 37 °C. This was followed by stimulation with the indicated substances and combinations thereof. After defined time points, the supernatants were collected and the remaining adherent cells were detached with Trypsin/EDTA, re-suspended in 1× DPBS (GibcoLife Technologies, Grand Island, NY, USA) and added to their respective supernatant. After centrifugation, the supernatant was aspirated, and the pelleted cells were incubated in an equal volume of Nicoletti Buffer for at least 20 min at 4 °C in the dark. Propidium iodide (PI) fluorescence signal was then detected in 5000 cells with a FACSCalibur flow cytometer using CELLQuest software. PI staining of DNA was used to discriminate the DNA content of permeabilized cells, whereby all cells with less than 2N DNA were defined as containing fragmented DNA and, therefore, as apoptotic. Treatment specific apoptosis rates were obtained by normalization of apoptosis rates in treated cells (experimental apoptosis) to spontaneous apoptosis rates in untreated cells with the following formula: specific apoptosis [%] = (experimental apoptosis [%] − spontaneous apoptosis [%])/(100 − spontaneous apoptosis [%]) × 100.

### 2.8. Pathology of the Original Tumour

Tumour material was obtained during the initial resection and tagged as specimen #322. The sample was split in two and either used as a base to obtain primary patient-derived cells (see above), or prepared and analysed by the Department of Neuropathology of Ulm University. The malignancy was confirmed, after histological staining, to be a MB by a certified pathologist. 

### 2.9. Characterisation of the Stem Cell-like Cells and Their Differentiated Progenies

The characterisation of the two different cell types of specimen #322 was performed by using three different Proteome Profiler™ Antibody Arrays (all from R&D Systems, Minneapolis, MN, USA): Human Phospho-Kinase Array Kit, Human Apoptosis Array Kit and Human XL Oncology Array Kit. The procedure was performed according to the manufacturer’s instructions. The average expression of two independent experiments was used to determine expression relative to the positive controls present on each membrane.

### 2.10. Chorioallantoic Membrane (CAM) Assay

A mixture of stem cell-like cells and differentiated cells were grown at a 20:80 ration on the CAM of fertilized chicken eggs, as previously described [21]. Briefly, 1 × 10^6^ cells in a 1:1 mixture of serum-free medium and Matrigel (BD Biosciences, Franklin Lakes, NJ, USA) were transplanted onto the CAM of one-week-old, fertilized eggs (LSL Rhein-Main, Dieburg, Germany). One day after seeding treatment was started, as the volume for topical application is limited in this assay (15 µL), higher concentrations of substances were used to compensate: 5 µM ABT-263, 2.5 nM vincristine or the combination of both. Treatment was administered directly onto the tumour. Four days after seeding, the tumour and surrounding CAM were extracted, embedded in paraffin and cut in sections with a thickness of 3 µm. The sections were stained with haematoxylin (Merck KGaA, Darmstadt, Germany) and eosin (Sigma-Aldrich, St. Louis, MO, USA), Ki-67 (1:100, #M7240, Dako Deutschland GmbH, Hamburg, Germany) or cleaved caspase 3 (1:20, #ab2302, Abcam plc, Cambridge, UK).

### 2.11. Statistical Analysis

The expected response to combination treatment was calculated as fractional response to drug A (Fa) + fractional response to drug B (Fb) − (Fa × Fb). Bliss analysis was conducted to detect synergistic (ration of actual total response and the expected total response > 1.1), additive (this 0.9 to 1.1) or antagonistic effects (quotient < 0.9).

Statistical significance was assessed by one-way ANOVA using Prism version 9.2.0 (GraphPad, La Jolla, CA, USA). A *p* ≤ 0.05 was considered statistically significant.

## 3. Results

### 3.1. Characterizing the Expression of the Bcl-2 Family in Medulloblastoma Cell Lines and Identifying Bcl-XL as Potential Therapeutic Target

**Figure 1:**
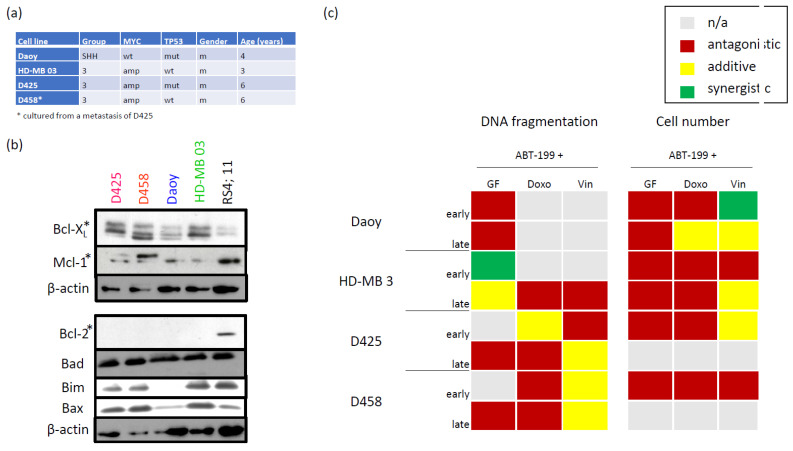
Bcl-XL, but not Bcl-2, is a promising potential therapeutic target in medulloblastoma (MB) cell lines. (**a**) Summary of cell line characteristics. Abbreviations: SHH, sonic hedgehog; wt, wild type; amp, amplified; mut, mutant; m, male. (**b**) Immunoblotting of Bcl-2 family protein expression in MB cell lines. Extracts of four different MB cell lines were subjected to immunoblotting for Bcl-2 family proteins. β-actin served as loading control and the leukaemia cell line RS4;11 served as positive control for Bcl-2 and Mcl-1 expression. Anti-apoptotic members of the Bcl-2 family are marked by an asterisk. (**c**) The inhibitor of Bcl-2, ABT-199, does not show a therapeutic effect on MB cell lines. Cells were stimulated with 10 nM ABT-199 in the presence of either reduced serum concentrations (to 1.5% FCS for Daoy cells or 5% FCS for all others), or the chemotherapeutic drug doxorubicin (5 nM for Daoy cells or 10 nM for all others), or vincristine (1 nM for Daoy cells or 0.5 nM for all others) for either 24 (early) or 96 (late) h, as described in the Materials and Methods section. Left: Cell death was assessed by the surrogate readout of DNA fragmentation, evaluated via flow cytometric analysis of PI-stained nuclei. Right: The number of live cells was counted using the CASY1 DT cell counter. Using Bliss analysis, synergistic (green), additive (yellow) or antagonistic (red) effects were determined, while grey indicates that Bliss analysis was mathematically not possible to perform. Shown in (**b**) is a representative example of two independent experiments, while (**c**) summarizes at least three independent experiments performed in triplicate.

While the modulation of the Bcl-2 family has shown great promise in haematological malignancies, such as Chronic Lymphocytic Leukaemia (CLL), Small Lymphocytic Lymphoma (SLL), or Acute Myeloid Leukaemia (AML), where Venetoclax (ABT-199) is an approved therapeutic option, the use of BH3 mimetics in solid tumours is less successful. To investigate the potential of this treatment approach in MB, the most common solid tumour in childhood, we first investigated the expression of key members of the Bcl-2 superfamily in four established MB cell lines (Figure 1a), using Acute Lymphoblastic Leukaemia (ALL) RS4;11 as an external reference (Figure 1b). The extremely low protein expression of Bcl-2 in all four MB cell lines, compared to the RS4;11 cells, suggests that ABT-199, an inhibitor specific to Bcl-2, should not be a potent sensitizer in this set of MB cells. This holds true with regards to both induction of apoptosis and general reduction in the number of live cells (Figure 1c). 

**Figure 2:**
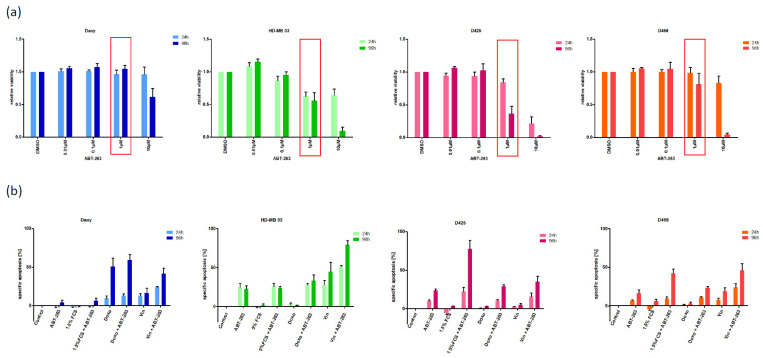

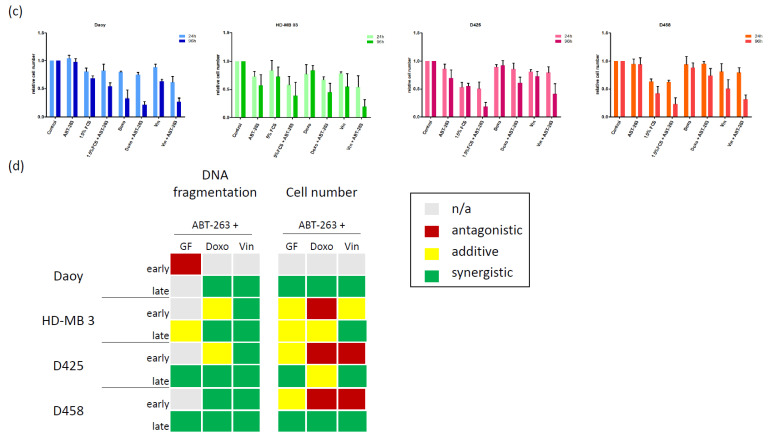
The Bcl-XL/Bcl-2 inhibitor ABT-263 shows therapeutic potential in medulloblastoma (MB) cell lines. (**a**) Monotreatment with ABT-263 affects the metabolic activity of MB cell lines. The MB cell lines were seeded and stimulated with a serial dilution of ABT-263 for 24 and 96 h. The relative viability of the respective populations was normalized to the solvent-treated control population. The boxed column represents the concentration selected for further experiments (1 µM). (**b**) Combining ABT-263 and different stressors can enhance apoptosis. The seeded cell lines were cultured in the presence of 1 µM ABT-263, reduced serum concentrations (to 1.5% FCS for Daoy cells or 5% FCS for all others), doxorubicin (5 nM for Daoy cells or 10 nM for all others) and vincristine (1 nM for Daoy cells or 0.5 nM for all others), as well as combinations of them (concentrations described in the Materials and Methods section). Percentage of specific DNA fragmentation was determined 24 and 96 h after stimulation by flow cytometric analysis of PI-stained nuclei. (**c**) Combining ABT-263 and different stressors can reduce living cell numbers. The experimental setup was similar to (**b**). This was followed by assessing the total living cell number 24 and 96 h after stimulation by CASY1 DT measurement. Data was normalized to a solvent-treated control population for each time point. (**d**) The inhibitor of Bcl-XL/Bcl-2, ABT-263, shows a therapeutic effect on MB cell lines. Similarly to the data shown in Figure 1b, the effects of combining ABT-263 with different stressors on apoptosis (left) and living cell number (right), as shown in (**b**,**c**), respectively (early, 24 h; late, 96 h), were analysed for synergism. Using Bliss analysis, synergistic (green), additive (yellow) or antagonistic (red) effects were determined, while grey indicates that Bliss analysis was mathematically not possible to perform. Shown in (**a**) are the mean and SD of at least three independent experiments with six individual data points. In (**b**) are the mean and SD of three independent experiments performed in triplicates, and in (**c**) are the mean and SD of at least four independent experiments performed in triplicates, while (**d**) is a mathematical summary of (**b**,**c**).

In contrast to Bcl-2, Bcl-XL is expressed at levels at least equal to those found in RS4;11 cells, suggesting that ABT-263 (Navitoclax), an inhibitor of both Bcl-2 and Bcl-XL, might have some therapeutic effect in these MB cell lines. Therefore, we next assessed the effect of various concentrations of ABT-263 on the metabolic activity of the cells (Figure 2a). ABT-263 exhibited a diverse effect on the four cell lines when used at low micromolar concentrations, a concentration range also identified in other malignant cells as effective [22]. Therefore, we decided to continue further experiments with a concentration of 1 µM, which showed little to no effect on the metabolic activity of two MB cell lines, while reducing it by approximately 50% in the other two (Figure 2a). Next, we looked at the effects on apoptosis (Figure 2b) and total live cell numbers (Figure 2c) when combining ABT-263 with additional stressors, namely reduction of nutrients, or doxorubicin and vincristine, both chemotherapeutic reagents commonly used in MB treatment. The analysis of the combinatory effect, as shown in Figure 2d, clearly indicates that the addition of stressors to the inhibitor of Bcl-XL frequently elicits a synergistic effect. Of note, even in the rare occasions when the combination is antagonistic, it is a weak antagonism, i.e., the combination is never less potent than the strongest individual treatment is on its own.

Taken together, these data suggest that modulation of the Bcl-2 superfamily, particularly Bcl-XL, can be of therapeutic benefits when devising novel treatment strategies for MB. However, because established cell lines are often considered poor surrogates for the patient situation [23], we next looked at the effect of BH3 mimetics in a different model system. 

### 3.2. Bcl-XL Modulation Is Also a Promising Therapeutic Approach in Primary Material

**Figure 3:**
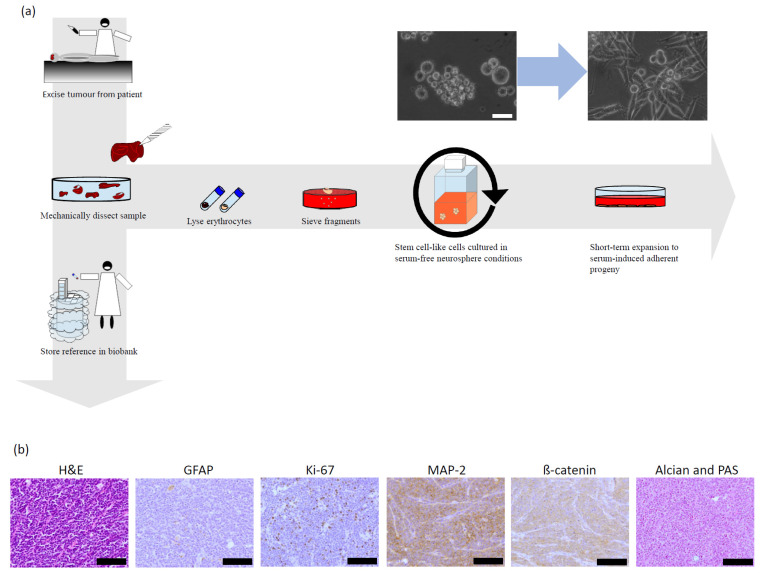

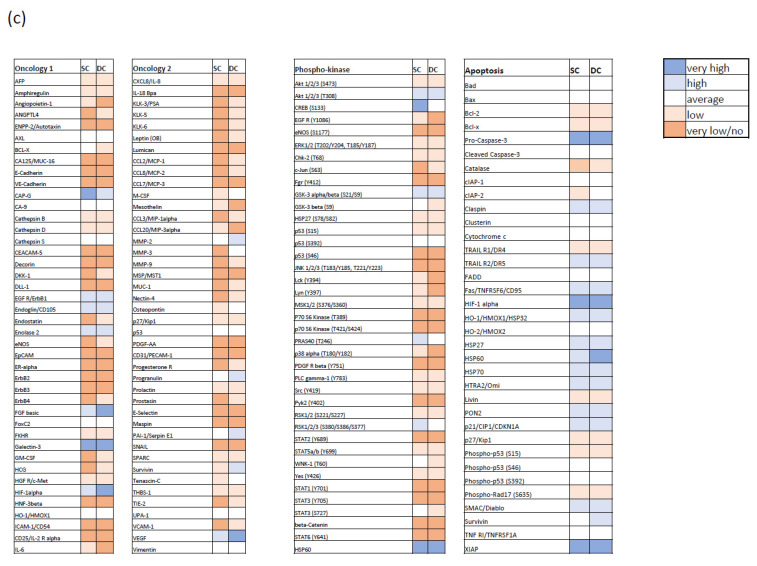
Characterisation of patient-derived medulloblastoma (MB) stem-like cells and short-term differentiated cells. (**a**) Schematic workflow of obtaining patient-derived MB cells. During therapeutically indicated surgery and following patient’s (or legal guardian’s) consent, a tumour sample surplus to histological characterisation and archiving was split into two. While one element was stored as reference material, the remaining tissue was further dissected and cleaned from blood cells. After several washes, fragments were sieved, and single cells/small cell clumps were cultured under non-adherent conditions in “stem cell” medium. Neurospheres that emerged after prolonged culturing were stored and characterised with reference to original tumour histology. Adherent, differentiated progeny were cultured from neurospheres via permitted adhesion and change of culture conditions, using “differentiation” medium. These differentiated populations were used for a maximum of ten generations to ensure genetic stability. Details are presented in the Materials and Methods section. Shown here are also exemplary pictures of the stem cell-like MB cells grown as free-flowing spheres and their adherently cultured differentiated progeny. Scale bar: 50 μm. (**b**) Neuropathology specimen of the patient’s tumour stained as indicated. The H&E staining shows small, round and densely packed cells. GFAP staining is negative as for most MB tumours, while a relatively high count of Ki-67 positive areas indicates an increased amount of cycling cells. MAP2 confirms the CNS origin of the tumour, while ß-catenin indicates that the tumour exhibits SHH activity. In addition, Alcian and PAS was used to detect polysaccharides, elevated in cancers. Taken together, these data suggest a medulloblastoma belonging to the SHH subgroup. Abbreviations: H&E—haematoxylin and eosin stain; GFAP—Glial Fibrillary Acidic Protein; Ki-67—Antigen KI-67; MAP2—Microtubule-Associated Protein 2; ß-catenin—βeta-catenin; Alcian & PAS—Alcian blue stain and Perodic acid–Schiff stain, SHH–sonic hedgehog. Scale bar: 200 µm. (**c**) Expression protein profile in MB stem cell-like cells and differentiated cells shown as a heat map. Protein extracts of a stem cell-like cell population (SC) and its differentiated progeny (DC) were subjected to immunoblotting for three different types of protein profile assays, as described in the Materials and Methods section. Following visualisation, membranes were densitometrically evaluated and bioinformatically processed. The map shows relative expression (blue, higher than average; red, lower than average; as described in the Materials and Methods section) and represents the mean of two independent experiments.

**Figure 4:**
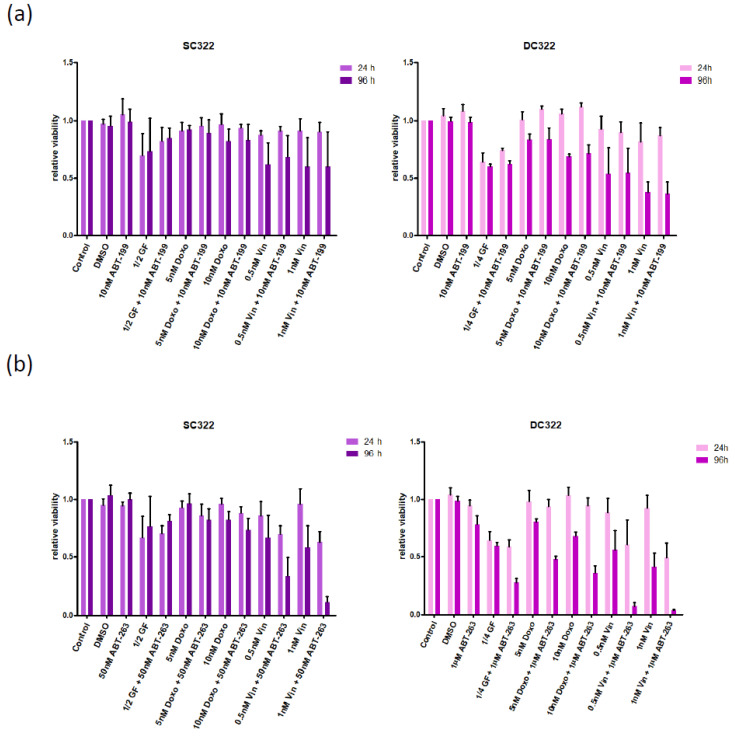

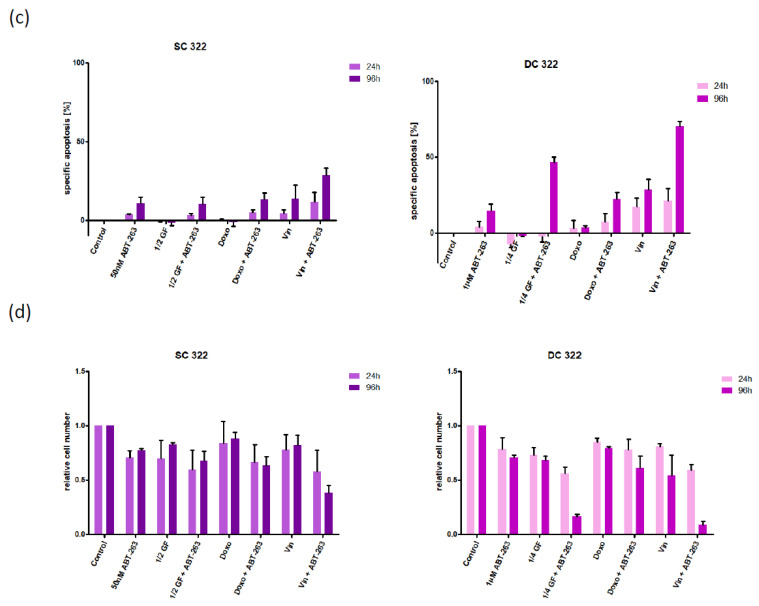

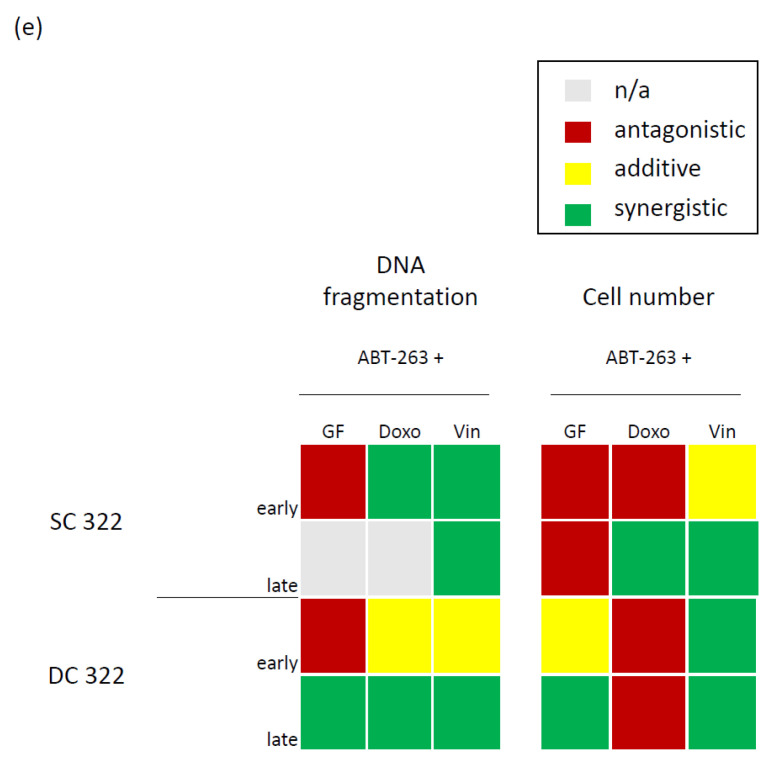
The Bcl-XL/Bcl-2 inhibitor ABT-263, but not the Bcl-2 inhibitor ABT-199, shows therapeutic potential in primary medulloblastoma (MB) cells. (**a**) The inhibitor of Bcl-2, ABT-199, does not show a therapeutic effect on the metabolism of primary MB cells. The stem cell-like cells (SC322, left) or their differentiated progeny (DC322, right) were cultured in the presence of ABT-199, reduced growth factor/serum concentrations, doxorubicin or two concentrations of vincristine as well as ABT-199 combined with the different stressors (concentrations as indicated). Metabolic activity was assessed after 24 and 96 h and normalized to the solvent-treated control population. (**b**) The inhibitor of Bcl-XL/Bcl-2, ABT-263, shows a therapeutic effect on the metabolism of primary MB cells. The experimental setup was similar to 4A. Metabolic activity was assessed after 24 and 96 h and normalized to the solvent-treated control population. (**c**) ABT-263 shows a therapeutic effect on cell death induction of primary MB cells. The stem cell-like cells (SC322, left) or their differentiated progeny (DC322, right) were cultured in the presence of 1 µM ABT-263, reduced growth factor/serum concentrations (as indicated), 5 nM doxorubicin or 0.5 nM vincristine as well as ABT-263 combined with the different stressors. Percentage of specific DNA fragmentation was determined 24 and 96 h after stimulation by flow cytometric analysis of PI-stained nuclei. (**d**) ABT-263 shows a therapeutic effect on living cell number of primary MB cells. The experimental setup was similar to 4C. The total living cell number was assessed 24 and 96 h after stimulation by CASY1 DT measurement and normalized to solvent-treated control population for each time point. (**e**) Combining ABT-263 and various stressors frequently elicits a synergistic effect in primary MB cells. The effects of combining ABT-263 with different stressors on apoptosis (left) and living cell number (right), as shown in (**c**) and (**d**), respectively (early, 24 h; late, 96 h), were analysed for synergism. Using Bliss analysis, synergistic (green), additive (yellow) or antagonistic (red) effects were determined, while grey indicates that Bliss analysis was mathematically not possible to perform. In (**a**–**d**) columns represent mean and SD of three independent experiments performed in triplicates.

Mimicking our well-established approach of obtaining stem cell-like cells (SCs) from primary glioblastoma samples [12,19], we isolated and cultured cells from a tumour specimen of an adolescent patient suspected to be harbouring a MB (Figure 3a) and confirmed by histopathology that the isolated tumour was a MB (Figure 3b). Protein expression and phosphorylation showed, as expected, slight differences in patterns obtained from SCs when compared to their differentiated progenies (DCs). Importantly, expression of key members of the Bcl-2 superfamily, including Bcl-2 itself, was clearly detectable (Figure 3c). Interestingly, despite the expression of Bcl-2, ABT-199, its specific inhibitor, had no discernible effect on the metabolic activity of SCs or DCs, alone or in combination with additional stressors (Figure 4a). In contrast, while ABT-263 did not have an effect on the metabolic activity of SCs and only marginally affected that of DCs, it clearly enhances the effect of additional stressors on the metabolic activity of both cell populations (Figure 4b). This enhancing effect is also seen with regards to apoptosis induction and total number of living cells (Figure 4c,d, respectively). Statistical analyses confirm that the observed effects are often of synergistic nature (Figure 4e). 

Taken together, this dataset suggests that, even when expressed relatively highly in primary patient material, i.e., to a similar extent as Bcl-XL, Bcl-2 is not an attractive target for MB therapy. While ABT-263, which in contrast to ABT-199 also inhibits Bcl-XL, shows little effect as monotherapy, it significantly synergises with additional stressors, such as nutrient deprivation and chemotherapeutic drugs, to kill two functionally distinct but genetically identical cell populations, SCs and DCs. As expected, SCs are more resistant to ABT-263 as well as the additional stressors, and the synergistic effect detected is less pronounced than the one observed in DCs. 

Importantly, in these experiments, SCs were cultured as spheres in suspension, while DCs were short-term differentiated to adherent cultures. To further approximate the in vivo situation, we added additional refinements to this model system.

### 3.3. Bcl-XL Modulation Also Show Effects in an Artificial Tumour Model

**Figure 5:**
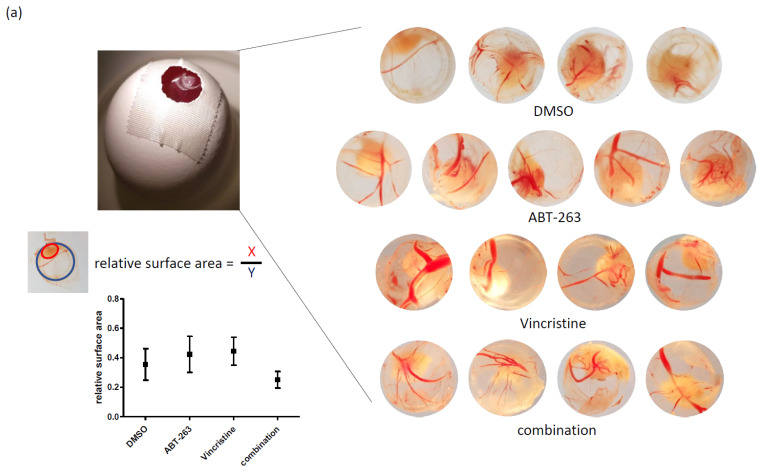

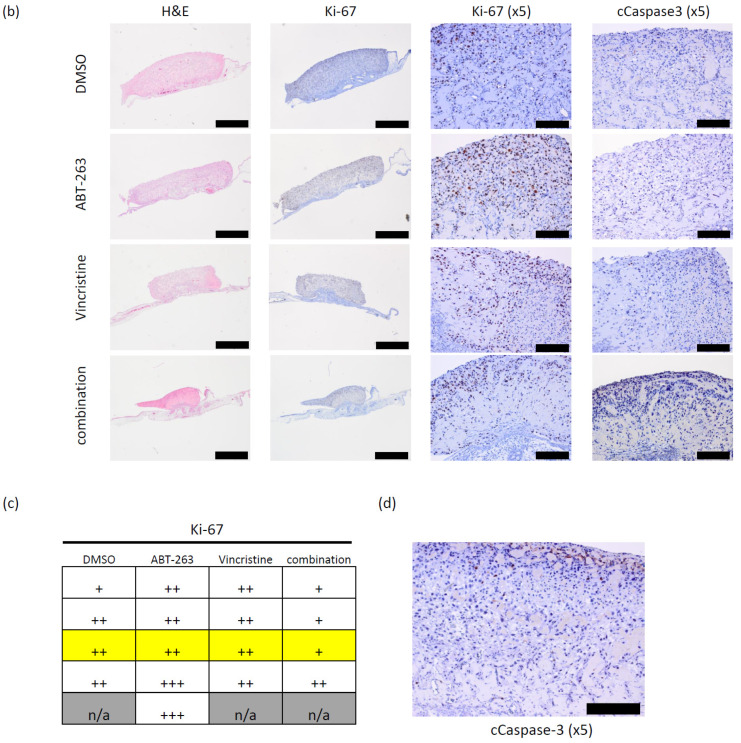
Combining the Bcl-XL/Bcl-2 inhibitor ABT-263 and vincristine shows superior therapeutic potential over single treatment in a chorioallantoic membrane (CAM) assay. (**a**) The surface area of double treated tumours appears to be reduced compared to mock or single treated entities. A 20:80 mixture of medulloblastoma stem cell-like cells and their differentiated progeny (SC/DC322) was seeded on the CAM of fertilized chicken eggs and were treated daily for four consecutive days with the indicated substances via local surface application (ABT-263: 5 µM, vincristine: 2.5 nM). After the aforementioned time span, the tumour surface area of all tumours (as shown on the right) was determined as indicated (bottom left). While a trend towards reduction is clearly visible, the reduction of surface area was statistically not significant, as determined by a one-way ANOVA test (*p*-value 0.0623). (**b**) Combining ABT-263 and vincristine has an effect on tumour morphology and cycling cells, but not necessarily on apoptosis induction. Shown are representative sections taken from the tumour centre of an individual tumour per treatment group, stained for haematoxylin and eosin (H&E), haematoxylin and Ki-67 (Ki-67) or haematoxylin and cleaved Caspase-3 (cCaspase-3). Scale: bars indicate 1000 µm, except in the columns labelled “(5x)”; here, bars indicate 200 µm. (**c**) Combining ABT-263 and vincristine increases the fraction of non-cycling cells. A histological evaluation of cells progressing through the cell cycle (Ki-67 positive) was performed. + indicates some positive cells clearly detectable, ++ indicates the majority of cells are positive, +++ almost all cells are positive; n/a, not available. Fields marked in yellow indicate the tumours depicted in (**b**). (**d**) High concentrations of ABT-263 can induce apoptosis. Depicted is a section of a tumour treated with ABT-263 only. Clearly visible is the positive staining for cleaved Caspase-3 (cCaspase-3) in the top layers of the section. These are the layers to which ABT-263 was directly applied and then allowed to diffuse through the tumour. Scale bar indicates 200 µm.

Next, we used the aforementioned (Figure 3b) experimental tumour system that combines SCs and DCs and used a basement membrane matrix to hold these cells together while transplanting them onto the chorioallantoic membrane (CAM) of a fertilized chick egg. This system gives the cells a more complex three-dimensional growth pattern, while concurrently providing a biological microenvironment. Following three days of treatment, only the tumours exposed to both ABT-263 and vincristine appear reduced in size (Figure 5a). Additionally, when looking at histological sections, vincristine alone seems to affect tumour morphology, as cells appear less tightly packed (Figure 5b). Whether this is due to increases in cell size, fibrosis or another phenomenon remains to be further investigated. Interestingly, the combination does not appear to greatly increase apoptosis induction as measured by Caspase 3 cleavage (Figure 5b, detailed analysis not shown). In contrast to this finding, the major effect the combination has in this setting appears to be anti-proliferative, as suggested by the Ki-67 staining (Figure 5b,c). This might suggest the possibility of a senescence-like phenotype being induced. Of note, sections of the tumour that came into direct contact with ABT-263, being locally and directly delivered, stained positive for cleaved Caspase 3 (Figure 5d), suggesting that ABT-263 can induce apoptosis in MB tumours when high enough local concentrations, here a 5 μM solution in a 15 μL volume, can be achieved.

## 4. Discussion

Medulloblastoma (MB), the most common primary brain tumour in children, presents as a diverse spectrum of distinct disease entities, of which, unfortunately, a considerable percentage remains refractory to therapeutic interventions [24]. In addition, it has been long known that even successful treatment of paediatric cancers is often associated with long-term sequelae [7,8]. To efficiently target the resistant subpopulation and minimize adverse consequences, we looked into potential candidates for an inducer/sensitizer approach; here, a sensitizer is utilized to break the intrinsic resistance to cell death, which characterizes cancer cells and allows for the reduction in the amount of cell death inducing therapy without reducing the therapeutic efficacy. One class of such sensitizers are pharmacological inhibitors of the Bcl-2 family, as these proteins have been implicated in mediating apoptosis resistance in a wide range of tumours [25]. 

When comparing the expression of Bcl-2 family members in four established MB cell lines with the expression pattern of the leukaemia cell line RS4,11, which has previously been described as amenable to therapeutic modulation of this protein family [26], we observed a low abundance of Bcl-2 protein, but high expression of Bcl-XL. We, therefore, postulated that Venetoclax (ABT-199), the inhibitor more specific for Bcl-2, would have only a neglectable effect on the MB cells, while Navitoclax (ABT-263), the inhibitor of Bcl-2 and, more importantly in this context, Bcl-XL, should be further explored. After verifying that, as expected, ABT-199 has only minor effects as a sensitizer for apoptosis, we concentrated on ABT-263.

Here, we could observe that the ABT-263 concentrations commonly described in the literature as effective (50 nM) [27] had, on their own, only minor effects on the metabolic activity of the MB cell lines. This is in contrast to some malignancies of the haemopoietic linage where the inhibitor alone can already efficiently kill the cells [28]. However, when used with additional stressors, such as various chemotherapeutics or reduction in nutrients, we could see that the inhibition of Bcl-XL synergizes with some of these stressors and, thus, leads to increased cell death and a more potent reduction in cell numbers. Interestingly, no clear pattern was discernible as to which cellular features predicted which stressor would synergize most efficiently with ABT-263.

Because only limited information can be obtained from established MB cell lines [23], we did not attempt to delineate the underlying molecular mechanisms, but considered these experiments as a proof of principle that MB potentially can be sensitized for apoptosis by inhibition of the Bcl-2 family and switched to a more relevant model, that of stem cell-like cells cultured as neurospheres and their short-term differentiated progeny. Interestingly, when characterizing these two genetically identical, but phenotypically and epigenetically distinct cell populations we found that here an expression of Bcl-2 was clearly detectable. However, when treating the cells with ABT-199 and additional stressors, we could find no synergism, suggesting that even if Bcl-2 is expressed in MB, it plays no pivotal role in mediating therapy resistance. In contrast, combination treatments with ABT-263 showed some effects. Here, we could observe a significantly higher rate of apoptosis and concurrent reduction of the living cell numbers due to the combined treatment of ABT-263 with chemotherapeutics, pointing clearly to a synergetic effect. ABT-263 sensitizes our primary MB cells to doxorubicin and potently to vincristine. 

This is in line with published data showing the increased resistance of stem cells, probably due to reduced proliferation rate [12]. The apoptosis rate is higher in differentiated cells than in stem cells. The most important reduction of cell populations may be observed after the cells are treated with vincristine combined with ABT-263. 

Finally, we further optimized the model by growing a 20:80 stem cell-like cells and differentiated progeny population as a three-dimensional tumour on the chorioallantoic membrane of a fertilized chick egg. In this in vivo-like setting, we treated the tumours with either DMSO, ABT-263, vincristine or a combination of the two active substances. Here, we were also able to observe the same trend as with the other experimental systems; tumours treated with the combination appeared smaller in size. Interestingly, no clear indications for increased apoptosis could be observed, instead the percentage of cells which stained positive for Ki-67, an indicator of an actively cycling cell, was markedly reduced. It appears that experimental tumours are reduced in size after combination treatment with ABT-263 and vincristine, due to a cytostatic effect and not due to increased cytotoxicity of the treatment. 

Vincristine is, of course, a well-known mitotic poison targeting the microtubules, and has long been associated with alterations in cell cycle and polyploidy [29]. It is possible that both the observed cell cycle arrest, as well as the minor induction of cell death, indicate that the combination treatment may induce senescence, a physiological, multifaceted process, which responds to excessive extracellular or intracellular stress by inducing a stable and generally irreversible cell cycle arrest [30,31]. Importantly, despite the permanent growth arrest, senescent cells remain viable and mostly resistant to apoptotic stimuli [32,33,34]. Moreover, the senescent state is accompanied by distinct phenotypic alterations, including the implementation of the senescence-associated secretory phenotype (SASP), which refers to the production of a complex mixture of secreted soluble and insoluble factors that can induce both beneficial as well as patho-physiological effects [31,35,36,37,38,39]. Intriguingly, the expression protein profile in MB cells reveals that stem cell-like cells already prior to treatment express a well-known pro-survival and pro-inflammatory cytokine interleukin (IL)-6 (Figure 3c), which is one of the most common SASP factors [40]. Hence, the presence of IL-6 in untreated cells could prime the cells for SASP and allow this phenotype to better spread through the cell population. It has recently been shown that senescent cells possess the ability to remodel and reorganize neighbouring cells and the microenvironment by activating various cell-surface receptors and corresponding signal transduction pathways through SASP [41]. Thus, further investigation is required to assess the potential of ABT-263 and vincristine to induce cellular senescence and the possible link to stem cell-like cells. Because there is no specific marker of senescence, future experiments will require the quantification of multiple factors and features such as the senescence-associated (SA)-ß-Galactosidase staining, p21 expression, and further assessment of the SASP factors [42,43,44]. 

ABT-263 had been investigated in clinical trials for the treatment of lymphoid malignancies or small cell lung cancer [45,46,47,48,49]. Importantly, from a therapeutic aspect, the use of ABT-263 is severely limited by the increased risk of haemocytopenia associated with its clinical application. ABT-263 is known to induce thrombopenia due to reduced platelet lifespan [50]; however, the next generation dual inhibitors of Bcl-XL and Bcl-2, such as AZD4320, have been reported to not induce dose-limiting thrombocytopenia [51] and, therefore, would be more suitable for a clinical application.

In conclusion, we believe that the modulation of the Bcl-XL protein might present a promising approach as part of a combination treatment for particular aggressive MB and suggest further evaluation of this therapeutic approach. While the Bcl-XL protein alone might not suffice to efficiently sensitise MB cells for conventional treatment, the use of inhibitors might be considered the first step towards improving the Bim/Bcl-XL ration with the use of Mcl-1 inhibitors as a further step towards treatment sensitisation.

## Data Availability

Data is contained within the article.
